# Genomic heterogeneity in pancreatic cancer organoids and its stability with culture

**DOI:** 10.1038/s41525-022-00342-9

**Published:** 2022-12-19

**Authors:** Olalekan H. Usman, Liting Zhang, Gengqiang Xie, Hemant M. Kocher, Chang-il Hwang, Yue Julia Wang, Xian Mallory, Jerome Irianto

**Affiliations:** 1 Department of Biomedical Sciences, College of Medicine, Florida State University, Tallahassee, FL 32306 USA; 2 Department of Computer Science, Florida State University, Tallahassee, FL 32306 USA; 3 Barts Cancer Institute, Queen Mary, John Vane Science Centre, University of London, Charterhouse Square, London, EC1M 6BQ UK; 4 Department of Microbiology and Molecular Genetics, University of California Davis, Davis, CA 95616 USA

**Keywords:** Cancer models, Cancer genomics, Genomic instability, DNA sequencing

## Abstract

The establishment of patient-derived pancreatic cancer organoid culture in recent years creates an exciting opportunity for researchers to perform a wide range of in vitro studies on a model that closely recapitulates the tumor. One of the outstanding question in pancreatic cancer biology is the causes and consequences of genomic heterogeneity observed in the disease. However, to use pancreatic cancer organoids as a model to study genomic variations, we need to first understand the degree of genomic heterogeneity and its stability within organoids. Here, we used single-cell whole-genome sequencing to investigate the genomic heterogeneity of two independent pancreatic cancer organoid lines, as well as their genomic stability with extended culture. Clonal populations with similar copy number profiles were observed within the organoids, and the proportion of these clones was shifted with extended culture, suggesting the growth advantage of some clones. However, sub-clonal genomic heterogeneity was also observed within each clonal population, indicating the genomic instability of the pancreatic cancer cells themselves. Furthermore, our transcriptomic analysis also revealed a positive correlation between copy number alterations and gene expression regulation, suggesting the “gene dosage” effect of these copy number alterations that translates to gene expression regulation.

## Introduction

Pancreatic ductal adenocarcinoma (PDAC) is the most common form of pancreatic cancer. With a 5-year survival rate of 11%, PDAC has one of the highest death rates among all cancers^1^. As part of the effort to improve the survival rate of PDAC and to develop more effective treatments, a better understanding of disease biology is urgently needed. In recent years, multiple studies have reported success in establishing PDAC organoid culture from patient tumors^2–8^. These organoids were shown to closely recapitulate the phenotype, the genotype, and the drug response of their in vivo tumor counterparts. PDAC organoid culture provides researchers with the opportunity to perform various in vitro studies on a model that closely represents the in vivo tumor, which in turn, will allow for a broader, deeper, and more accurate understanding of the disease. One characteristic of PDAC is its genomic heterogeneity in both primary tumors and metastatic lesions^9–16^. However, the causes and consequences of the observed genomic heterogeneity are largely unknown. A better understanding of genomic heterogeneity will help us elucidate both the etiology of the disease and its progression. PDAC organoid culture provides the opportunity to perform high-resolution genotyping and detailed mechanistic studies. Moreover, sample purity has been a significant issue in tissue-based genomic studies, where low purity has been shown to compromise the accuracy of the genomic data^17^. PDAC organoid culture, on the other hand, yields samples with the highest cancer cell purity.

To use PDAC organoids as a model to study genomic variations, however, we first need to understand the baseline genomic heterogeneity and stability of PDAC organoids. Without this understanding, it will be challenging to accurately interpret the genomic data after specific genetic modulations, treatments, or other perturbations. One of the pioneering studies by the Tuveson group used karyotyping to show the ploidy shifts within the extended PDAC organoid culture^3^. A following-up study by the same group derived single-cell clones from the organoid cultures^18^ and showed sub-clonal copy number variations (CNVs) between the clones, indicating genomic heterogeneity and instability within the PDAC organoids. Here, using single-cell whole-genome sequencing (scWGS) on two independent lines of PDAC organoids, we significantly increased the cellular resolution of organoid genotyping compared with previous studies. We observed genomic heterogeneity, in the form of chromosome copy number alterations, within both lines of PDAC organoids. Furthermore, we identified clonal and sub-clonal populations and elucidated their correlations through a phylogenetic tree analysis. Lastly, our transcriptomic analysis also revealed the functionality of these copy number alterations, suggesting a “gene dosage” effect^19–21^ of these copy number alterations that translates to gene expression regulation.

## Results

### Copy number alterations in two lines of patient-derived PDAC organoids

**Fig. 1:**
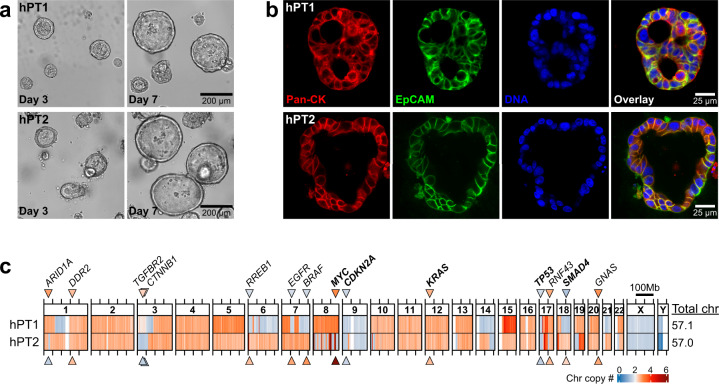
Chromosome copy number alterations in patient-derived pancreatic ductal adenocarcinoma (PDAC) organoids. **a** Representative images of primary tumor-derived PDAC organoids, hPT1 and hPT2, from two independent patients, showing the growth of the organoids over 4 days (bar = 200 µm). **b** Confocal sections of hPT1 and hPT2 organoids immunostained for cytokeratin (red) and EpCAM (green), both are markers of epithelial and PDAC cells (bar = 25 µm). **c** Genomic DNA of passage 3 of hPT1 and passage 6 of hPT2 was subjected to SNPa, and the resulting data was used to derive chromosome copy numbers of the organoids within 1 Mb binning windows. The heatmaps illustrate chromosome copy numbers across the whole genome, where blue indicates chromosome loss (<2) and red indicates chromosome gain (>2). The copy number of the frequently mutated genes in PDAC is indicated by the color of the corresponding arrowheads. The total chromosome number was estimated from the summation of the average copy number of each chromosome. Chromosome copy number alterations were observed across the genome, and both organoids had sub-triploid ploidy of ~57 chromosomes.

Two patient-derived primary tumor PDAC organoids (hPT1 and hPT2) were acquired and expanded for this study (Fig. 1a). The doubling time of both organoids was about 7 days, hence the organoids were passaged at a 1:2 ratio weekly. Immunostaining clearly showed that the organoids were positive for both cytokeratin and epithelial cell adhesion molecule (EpCAM), reflecting their epithelial origin (Fig. 1b). To analyze their chromosome copy number profiles, the genomic DNA of both hPT1 and hPT2 organoids was subjected to single-nucleotide polymorphism microarray (SNPa), and the data was used to derive their copy number profiles at 1 Mb resolution (Fig. 1c). We previously benchmarked this method and demonstrated that SNPa produced similar results as the comparative genome hybridization array^22^, which is the gold standard for chromosome copy number profile measurements^23^. Chromosome copy number alterations were observed across the genome of both hPT1 and hPT2 (Fig. 1c). The total chromosome number was estimated from the summation of the average copy number of each chromosome. In general, copy gains were more prevalent than losses, resulting in a sub-triploid ploidy of 57.1 and 57 chromosomes for hPT1 and hPT2, respectively. Additionally, we observed unusually high copy number alterations in chromosome 8 of hPT2, where the copy number appeared to oscillate between losses and very high gains. This pattern is in line with the typical signatures of chromothripsis, as reported in numerous cancers, including PDAC^24,25^. The role of chromothripsis in PDAC metastatic progression was suggested through the amplification of *MYC* at chromosome 8^26^. Indeed, the copy number of *MYC* in hPT2 is higher than in hPT1 (Fig. 1c).

The copy number of some genes, including the four most common driver mutation genes^27^: *KRAS*, *CDKN2A*, *TP53*, and *SMAD4*, are also frequently altered in PDAC^28^. In both hPT1 and hPT2 (Fig. 1c), the oncogene *KRAS* is amplified, while the tumor suppressors *CDKN2A* and *TP53* have reduced copy numbers. Interestingly, the copy loss of the tumor suppressor *SMAD4* was only observed in hPT1, suggesting a potential phenotypic distinction between hPT1 and hPT2. Differences in copy number alterations were also observed in the other frequently altered genes, including *ARID1A*, *TGFBR2*, *CTNNB1*, *RREB1*, *EGFR*, and *BRAF*.

### Genomic heterogeneity in PDAC organoids and genomic shift with prolonged culture

SNPa provides a bulk-level overview of the average copy numbers of all the cells in the PDAC organoid. To improve our cellular resolution in understanding the genomic heterogeneity of PDAC organoids, we performed scWGS on both hPT1 and hPT2. We further examined each organoid line at two passage numbers to gain insights into the genomic stability of organoid culture.

**Fig. 2:**
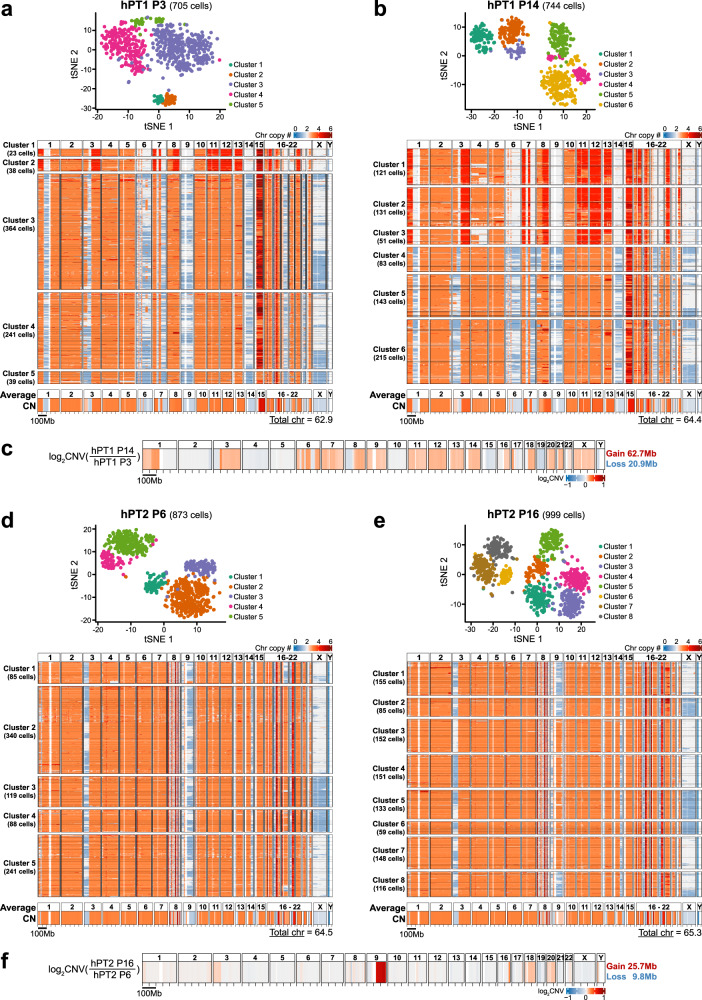
Single-cell whole-genome sequencing reveals genomic heterogeneity within PDAC organoids and the genomic shift with culture. **a** From passage 3 of hPT1 (hPT1 P3), we acquired scWGS data of 705 cells. The single-cell copy number data derived from the scWGS were subjected to t-SNE analysis, followed by spectral clustering to derive the number of clusters within the sample. The spectral clustering of the hPT1 P3 t-SNE plot resulted in five distinctive clusters. The single-cell chromosome copy number profiles of each cluster were plotted in the form of heatmaps, with the resolution of a 5 Mb binning window. Genomic heterogeneity is evident within the population. Clusters 1 and 2 show a distinct group of cells (totaling 61 cells) with partial chromosome copy gains in chromosomes 1, 3, 7, 8, and 16, in addition to whole chromosome copy gains in chromosomes 11, 12, 13, and 20. Genomic heterogeneity can also be observed in the rest of the cells (Clusters 3–5, totaling 644 cells) through the intermittent copy losses within chromosomes 1, 3, 6, 7, 9, 14, 18, 21, and X, as well as intermittent copy gains within chromosomes 3 and 15. The bottom heatmap shows the averaged copy number from the 705 hPT1 P3 cells. The copy number pattern across the genome is in accordance with the hPT1 copy number profile derived from the SNPa in Fig. 1c. The total number of chromosomes was estimated to be 62.9, which is slightly higher than the one estimated by the SNPa, but both of them are still within the sub-triploid ploidy range. **b** From passage 14 of hPT1 organoids (hPT1 P14), we acquired scWGS data of 744 cells. The spectral clustering of the hPT1 P14 t-SNE plot resulted in six distinctive clusters. The copy number profiles of hPT1 P14 Clusters 1–3 resemble the cells in hPT1 P3 Clusters 1 and 2, however, the proportion of these cells increases within the hPT1 P14 population (totaling 303 cells), suggesting a clonal population expansion with the extended culture of 11 passages. Genomic heterogeneity within the hPT1 P14 Clusters 1–3 cells can be observed in chromosomes 1, 4, 5, 11, 13, 16, and 22. The copy number profiles of the rest of the cells (Clusters 4–6, totaling 441 cells) look similar to the hPT1 P3 Clusters 3–5 cells, except for the partial loss of chromosome 8 within all of the hPT1 P14 Cluster 4 cells and some cells in Clusters 5 and 6. The bottom heatmap shows the averaged copy number from the 744 hPT1 P14 cells. The total number of chromosomes was estimated to be 64.4, which is higher than hPT1 PT3, indicating copy number gains with extended culture. **c** The CNV between hPT1 P3 and hPT1 P14 was derived from the averaged copy number data. Chromosome copy gains were observed in chromosomes 1, 3, 6, 7, 8, 9, 11, 12, 13, 14, 16, 17, 18, 20, 21, and X, which correspond to the clonal expansion in hPT1 P14 Cluster 1–3. All combined, the extended culture resulted in approximately 62.7 Mb copy gains and 20.9 Mb copy losses. **d** From passage 6 of hPT2 (hPT2 P6), we acquired scWGS data of 873 cells. The spectral clustering of the hPT2 P6 t-SNE plot resulted in five distinctive clusters. Similar to hPT1 organoids, genomic heterogeneity can be observed within the hPT2 population. In contrast to the other clusters, the cells within Cluster 1 have intermittent copy losses in chromosome 18, instead of copy gains. Eleven cells within Cluster 1 have uniquely partial gains in chromosome 9 and partial diploid in chromosome 4. The copy number profiles of the rest of the cells (Clusters 2–5, totaling 788 cells) are generally similar to each other, with some level of genomic heterogeneity in the form of intermittent copy losses within chromosomes 3, 9, 13, 14, 17, 19, and X. The bottom heatmap shows the averaged copy number from the 873 hPT2 P6 cells, which is in accordance with the hPT2 copy number profile derived from the SNPa in Fig. 1c. The total number of chromosomes was estimated to be 64.5. **e** From passage 16 of hPT2 organoids (hPT2 P16), we acquired scWGS data of 999 cells. The spectral clustering of the hPT2 P16 t-SNE plot resulted in eight distinctive clusters. The copy number profile of hPT2 P16 Clusters 1, 2, and 8 (totaling 356 cells) resemble the cells in hPT2 P6 Clusters 2–5, with the addition of copy gains in chromosome 20 in some cells. The rest of the cells in Clusters 3–7 (totaling 643 cells) have partial copy gains in chromosome 9, resembling the 11 cells in hPT2 P6 Cluster 1, again suggesting a clonal population expansion with the extended culture. However, in contrast to the cells in hPT2 P6, these 643 cells also have copy gains in chromosomes 4 and 18. The bottom heatmap shows the averaged copy number from the 999 hPT2 P16 cells. The total number of chromosomes was estimated to be 65.3, which is higher than hPT2 P6, indicating copy number gains with extended culture. **f** The CNV between hPT2 P6 and hPT2 P16 was derived from the averaged copy number data. Significant chromosome copy gains were observed within chromosome 9, which were caused by the clonal expansion in hPT2 P16 Clusters 3–7. Other copy gains were also observed in chromosomes 3, 16, 18, and 20. The extended culture resulted in approximately 25.7 Mb copy gains and 9.8 Mb copy losses.

From passage 3 of hPT1 organoids (hPT1 P3), we acquired scWGS data from 705 cells. We derived the copy number profile of each cell at 5 Mb resolution. Of note, the resolution of the copy number profile from scWGS is lower than that from SNPa. Averaging the sequencing data at a bigger 5 Mb window is necessary due to the relatively shallow sequencing depth, ranging between 0.015× and 0.042× for the scWGS samples. To systematically analyze the genomic heterogeneity within each sample, we performed t-SNE analysis on the single-cell copy number profiles, followed by spectral clustering to derive clusters of closely related cells. The spectral clustering of the hPT1 P3 t-SNE plot resulted in five clusters (Fig. 2a). Genomic heterogeneity was clearly observed within the hPT1 P3 population. Specifically, two distinct groups of cells were observed. The first group of cells was contained in Clusters 1 and 2, totaling 61 cells. Compared to the rest of the population, the cells in Clusters 1 and 2 had partial copy gains in chromosomes 1, 3, 7, 8, and 16, and whole chromosome copy gains in chromosomes 11, 12, 13, and 20. The second group of cells was contained in Clusters 3–5, totaling 644 cells. Genomic heterogeneity within this group was observed through the intermittent copy losses across the cells within chromosomes 1, 3, 6, 7, 9, 14, 18, 21, and X, as well as copy gains within chromosomes 3, 8, 13, and 15. When we averaged the single-cell copy number data from the 705 hPT1 P3 cells (Fig. 2a bottom), the resulting copy number profile is in accordance with the SNPa copy number profile of hPT1 (Fig. 1c), confirming the concordance with the scWGS method. One difference between the aggregated scWGS profile and the SNPa profile is that the estimated number of chromosomes from the scWGS data is higher than the one estimated by SNPa. This slight discrepancy may be because they were derived from different methods and/or because of the different copy number resolutions, 5 Mb for scWGS and 1 Mb for SNPa. Nevertheless, the higher scWGS estimate is still within the sub-triploid ploidy range.

Next, we performed scWGS analysis on passage 14 of hPT1 organoids (hPT1 P14). As we passaged the organoids weekly, hPT1 P3 was cultured for 11 weeks to reach hPT1 P14. At P14, we acquired scWGS data from 744 cells. The spectral clustering of the hPT1 P14 t-SNE plot resulted in six clusters (Fig. 2b). From the copy number profiles, two distinct groups of cells were also observed in hPT1 P14. The copy number profiles of the first group of cells in Clusters 1–3 resembled the cells in hPT1 P3 Clusters 1 and 2. However, the proportion of these cells in hPT1 P14 grew to ~40.7% (303 cells out of 744 cells) from ~8.7% in hPT1 P3, suggesting a clonal population expansion with extended culture. Genomic heterogeneity within the hPT1 P14 Clusters 1–3 cells was observed within chromosomes 1, 4, 5, 11, 13, 16, and 22, which were absent in hPT1 P3 Clusters 1 and 2 cells. The copy number profiles of the 441 cells in hPT1 P14 Clusters 4–6 resembled the hPT1 P3 Clusters 3–5 cells, with the exception of the partial loss of chromosome 8 within all of the hPT1 P14 Cluster 4 cells and some of the cells in Clusters 5 and 6. In all, the total number of chromosomes was estimated to be 64.4, which is slightly higher than in the hPT1 P3, indicating copy number gains with the extended culture. Indeed, when we derived the CNV between hPT1 P3 and P14 from the averaged copy number profile (Fig. 2c), 62.7 Mb copy gains were observed across chromosomes 1, 3, 6, 7, 8, 9, 11, 12, 13, 14, 16, 17, 18, 20, 21, and X and 20.9 Mb copy losses were observed in other chromosomes. Most of the observed genomic shifts correspond to the clonal expansion in hPT1 P14 Clusters 1–3.

Similar scWGS analyses were performed for the hPT2 organoids. From passage 6 of hPT2 organoids (hPT2 P6), we acquired scWGS data from 873 cells. The spectral clustering of the hPT2 P6 t-SNE plot resulted in five clusters (Fig. 2d). Three distinct groups of cells were observed within the single-cell copy number profiles of hPT2 P6 cells. The differences between these three groups of cells revolved around chromosomes 4, 9, and 18. First, the 85 cells within Cluster 1 had intermittent copy losses in chromosome 18, instead of the copy gains that were observed in the other clusters. Second, within Cluster 1, 11 cells had uniquely partial gains in chromosome 9, accompanied by partial diploid in chromosome 4. The rest of the 788 cells in Clusters 2–5 had copy gains in chromosomes 4 and 18, and intermittent losses in chromosome 9. Genomic heterogeneity in these 788 cells was observed in the form of intermittent copy losses within chromosomes 3, 9, 13, 14, 17, 19, and X. The unusual oscillating copy number profile in chromosome 8, as illustrated in Fig. 1c, was also observed under scWGS in all cells. Furthermore, the averaged copy number profile of hPT2 P6 under scWGS (Fig. 2d bottom) also highly resembled the SNPa copy number profile of hPT2 P6 in Fig. 1c. The total number of chromosomes for the hPT2 P6 cells was estimated to be 64.5, again, slightly higher than the number estimated by the SNPa copy number profile (Fig. 1c). After an extended culture for 10 additional passages, the scWGS data of hPT2 P16 revealed a clonal expansion of the cells that had partial copy gains in chromosome 9, overtaking the population majority from ~1.3% in hPT2 P6 to ~64.4% in hPT2 P16 (Clusters 3–7, totaling 643 cells out of 999 cells, Fig. 2e), again suggesting a clonal population expansion with extended culture. However, in contrast to hPT2 P6, these 643 hPT2 P16 cells had copy gains in both chromosomes 4 and 18. The rest of the 356 cells in hPT2 P16 Clusters 1, 2, and 8 resembled the cells in hPT2 P6 Clusters 2–5, with the addition of copy gains in chromosome 20 in some cells. From the averaged copy number profile, we estimated the chromosome number of hPT2 P16 to be 65.3, which was slightly higher than the number estimated for hPT2 P6, again suggesting a gain of copy number with extended culture. Indeed, from the CNV analysis between hPT2 P6 and P16 (Fig. 2f), significant copy gains were observed within chromosome 9, which were caused by the clonal expansion in hPT2 P16 Clusters 3–7, and also other copy gains in chromosomes 3, 16, 18, and 20. All combined, the extended culture resulted in approximately 25.7 Mb of copy gains and 9.8 Mb of copy losses.

**Fig. 3:**
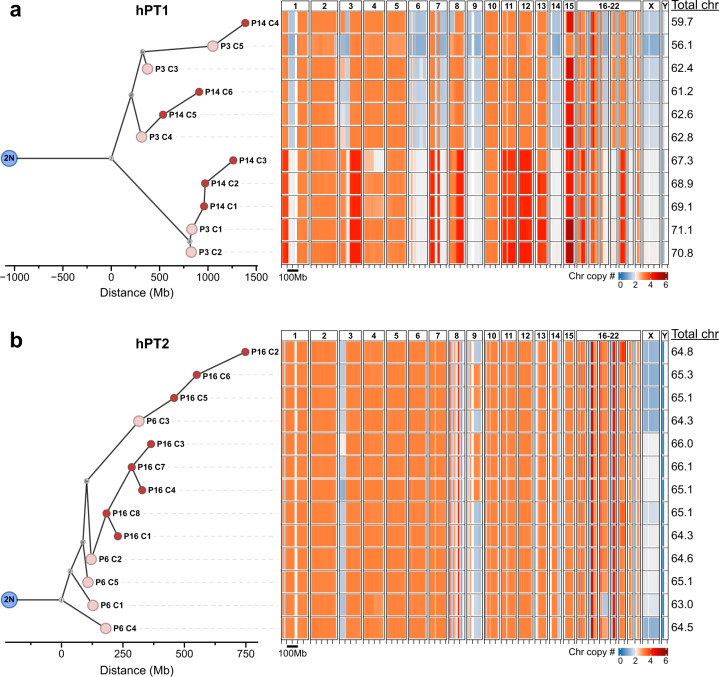
Phylogenetic tree analysis reveals the correlation between the identified sub-population clusters and enables the quantification of genomic heterogeneity. The single-cell chromosome copy number data of the clusters identified in Fig. 2 were averaged and used to derive phylogenetic trees for hPT1 and hPT2. The maximum parsimony method was used to build the trees from the early passage clusters, followed by the minimal distance pairings of the late passage clusters. These trees reveal the correlation between the clusters of different culture periods and quantify the copy number differences between the clusters, as a measure of genomic heterogeneity. **a** hPT1 phylogenetic tree reveals two distinct groups of clusters that are separated by ~1500 Mb of copy number differences. Closer to the diploid (2 N) root, the first group includes hPT1 P3 Clusters 3–5 (P3 C3–5), and hPT1 P14 Clusters 4–6 (P14 C4–6). P14 C5–6 are paired to P3 C4, while P14 C4 is paired to P3 C5. The estimated chromosome number ranges from 56.1 to 62.8. The other group of clusters, spread within ~500 Mb of copy number differences, includes P3 C1–2, and P14 C1–3, which are paired with P3 C1. The estimated chromosome number ranges from 67.3 to 71.1. **b** The clusters in the hPT2 phylogenetic tree are distributed within ~750 Mb of copy number differences, which is smaller than the hPT1 tree, suggesting lower genomic heterogeneity within the hPT2 population. The hPT2 P6 Clusters 1–5 (P6 C1–5) are spread within ~300 Mb and are closely related to each other. hPT2 P16 Clusters 1, 3, 4, 7, and 8 (P16 C1, C3–4, C7–8) are paired to P6 C2, while P16 C2 and C5–6 are paired to P6 C3. The estimated chromosome number ranges from 63 to 66.1.

Next, to investigate the evolutionary distance between the clusters of cells, the copy number profiles of each cluster were averaged and used to derive phylogenetic trees for hPT1 and hPT2. The maximum parsimony method^29^ was used to build the trees from the early passage clusters, followed by the minimal distance pairings of the late passage clusters. This approach enabled us to reveal the correlation between the clusters of cells from different culture periods and to quantify the copy number differences between the clusters, as a measure of genomic heterogeneity. The hPT1 phylogenetic tree revealed two distinct groups of clusters that were separated by ~1500 Mb of copy number differences (Fig. 3a). Closer to the diploid root, the first group includes hPT1 P3 Clusters 3–5 (P3 C3–5), and hPT1 P14 Clusters 4–6 (P14 C4–6). P14 C5–6 are paired to P3 C4, while P14 C4 is paired to P3 C5. These pairings indicate the correlation between the late passage clusters to the early ones, and again confirm the existence of the same clonal population across the extended culture. The estimated chromosome number ranges from 56.1 to 62.8. The other group of clusters includes P3 C1–2, and P14 C1–3, which are paired to P3 C1. The estimated chromosome number ranges from 67.3 to 71.1. Unlike the first group, the clusters in this second group spanned within ~500 Mb of copy number differences, indicating a lower level of genomic heterogeneity within these clusters.

The clusters in the hPT2 phylogenetic tree were distributed within ~750 Mb of copy number differences, which was smaller than the hPT1 tree, suggesting lower genomic heterogeneity within the hPT2 population (Fig. 3b). The hPT2 P6 Clusters 1–5 (P6 C1–5) are spread within ~300 Mb and are closely related to each other. hPT2 P16 Clusters 1, 3, 4, 7, and 8 (P16 C1, C3–4, C7–8) are paired to P6 C2, while P16 C2 and C5–6 are paired to P6 C3. The estimated chromosome number ranges from 63 to 66.1. The copy number changes associated with each edge of the phylogenetic trees can be found in Supplementary Dataset 1.

In addition to the phylogenetic tree analysis, we also performed an unsupervised hierarchical clustering analysis of the sub-population clusters of hPT1 and hPT2 (Supplementary Fig. 1), where the distance between clusters was calculated using the Canberra distance method. Indeed, the correlation between the clusters identified by the hierarchical clustering highly resembles the correlations derived from the phylogenetic tree, confirming the findings of the phylogenetic analysis.

### Copy number alterations in PDAC organoids translate to transcript regulation

**Fig. 4:**
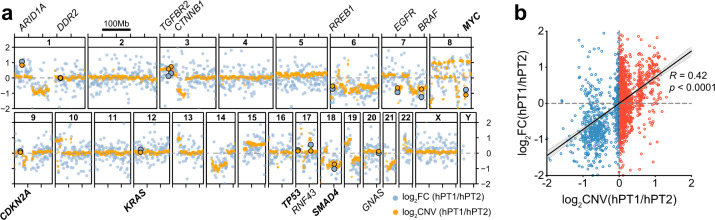
Copy number alterations translate to transcript regulations. **a** Both hPT1 P3 and hPT2 P6 organoids were subjected to RNA-seq. The gene expression fold change (log_2_FC, blue) between the two was averaged for every 1 Mb region across the whole genome. The plot shows good accordance between the gene expression fold change and the copy number variations (log_2_CNV from SNPa copy number profiles, orange) of the two organoids, especially in chromosomes 1, 3, 6, 7, 10, 14, 15, 19, and 21. The general agreements between log_2_FC and log_2_CNV are also observed for the commonly mutated genes in PDAC. **b** The CNV of each 1 Mb region was plotted against the corresponding gene expression fold change, resulting in a positive correlation between CNV and gene expression fold change. The correlation has a Pearson correlation coefficient (*R*) of 0.42, which is statistically significant, with *p* < 0.0001.

To investigate the functionality of these copy number alterations, the transcriptomes of hPT1 and hPT2 were analyzed through RNA-seq. The gene expression fold change between the two organoids was quantified and its profile across the whole genome was averaged at every 1 Mb. CNVs between hPT1 and hPT2 were derived from the SNPa copy number profiles that also have 1 Mb resolution. The matching resolution enables the comparison between gene expression regulation and the CNVs between the two organoids (Fig. 4a). The variation within the gene expression regulation profile is much higher than the CNV, which may be due to the trans-regulation of transcription^30^. However, the agreement between the CNV and gene expression regulation profile can be observed across the whole genome, especially where CNVs occur, such as in chromosomes 1, 3, 6, 7, 10, 14, 15, and 21. Indeed, such agreement was also observed in some of the frequently altered genes in PDAC^28^, including *ARID1A*, *CTNNB1*, *RREB1*, *EGFR*, *BRAF*, *MYC*, and *SMAD4*. CNVs of hPT1 and hPT2 positively correlated to the gene expression regulation between the two organoids, with a statistically significant Pearson correlation coefficient of 0.42 (Fig. 4b), suggesting “gene dosage” effects^19–21^ and functionality of chromosome copy number changes.

Gene set enrichment analysis (GSEA)^31,32^ of the regulated genes identified several “hallmark” gene sets^33^ that are significantly enriched in hPT1 and hPT2 (Supplementary Fig. 2). A couple of cell cycle-related gene sets (E2F targets and G2M checkpoint gene sets) were enriched in hPT1 (Supplementary Fig. 2A). Interestingly, SMAD4 has been shown to suppress cell proliferation through the TGF-β pathway^34^ and *SMAD4* deficiency in the *KRAS G12D* PDAC mouse model promotes cell proliferation^35^. These previous findings suggest the possibility that the enrichment of the cell cycle-related gene sets might be driven by the copy loss and downregulation of *SMAD4* in hPT1 (Figs. 1c and 4a). Moreover, the gene set that is upregulated by the activation of WNT signaling through the accumulation of CTNNB1 (WNT-beta catenin signaling gene set) is also enriched in hPT1, which might be driven by the copy gain and upregulation of *CTNNB1* in hPT1 (Figs. 1c and 4a). Both of these enrichments in hPT1 suggest the functional impact of chromosome copy number changes on cellular phenotypes. Furthermore, the genes that are upregulated in hPT1 also drive the enrichment of the epithelial-mesenchymal transition gene set, metabolism-related gene sets (hypoxia, glycolysis, cholesterol homeostasis, oxidative phosphorylation, reactive oxygen species pathway, and bile acid metabolism gene sets), and the genes that are upregulated through activation of mTORC1 complex (mTORC1 signaling gene set). Meanwhile, the upregulated genes in hPT2 resulted in the enrichment of genes that are important for cell polarity (apical surface gene set), immune response-related genes (allograft rejection and complement gene sets), and genes that are downregulated by KRAS activation (KRAS signaling down gene set) (Supplementary Fig. 2b).

## Discussion

In this study, we used two independent lines of patient-derived PDAC organoids, hPT1 and hPT2, to elucidate genomic heterogeneity within the organoid cultures. Chromosome copy number alterations were evident across the genome of both organoids (Fig. 1c), and copy number gains were more prevalent than the losses, resulting in a sub-triploid ploidy. Such copy number alterations were also reported in previous PDAC genomic studies involving both tumor tissues^9,10^ and organoids^3,5,8,18,36^. Additionally, by performing karyotyping, one of the pioneering studies that derived the PDAC organoid^3^ also showed ploidy heterogeneity and shift within the organoid culture. Here, we further investigated this genomic heterogeneity within PDAC organoid cultures and their genomic stability with extended culture by using scWGS (Figs. 2 and 3). With this technology, we can derive high-resolution copy number profiles at single-cell resolution. Clonal populations with similar copy number profiles were observed within the organoids, and the proportion of these clones was shifted with extended culture, suggesting the growth advantage of some clones. However, sub-clonal genomic heterogeneity was also observed within each clonal population, indicating the genomic instability of the PDAC cells themselves. Considering the functionality of these copy number alterations in gene expression regulation (Fig. 4), we speculate that the observed genomic heterogeneity here should translate to a population of cells with heterogeneous phenotypes. Indeed, such transcriptomic heterogeneity was also reported by a single-cell RNA-seq study on PDAC organoids^37^.

On a side note, it is important to recognize several limitations in the PDAC organoid model when interpreting the findings in this study. First, the PDAC organoids were maintained in a culture media that is supplemented with growth factors and various inhibitors, and they are also cultured within Matrigel, which is comprised of various extracellular matrix proteins. These culture conditions will potentially select a subset of cells that are more adapted to this culture system, resulting in a growth advantage for these cells. Similar selection limitation was also reported in patient-derived xenograft mouse models^38^. Hence, it is important to note that the genomic heterogeneity and clonal shift observed in this study might be impacted by the culture condition itself, in addition to the intrinsic genomic instability. Secondly, passaging the organoids weekly at a 1:2 ratio might also introduce a technical limitation to the organoid culture. There is a possibility for a rare population to be passaged into a Matrigel dome while absent in the other domes. In such a case, we will miss that rare population if we sample the Matrigel domes without that rare population for scWGS. Hence, our current passaging procedure with mechanical dissociation and a 1:2 ratio may potentially suppress the distribution of rare populations or low-abundance cells.

All combined, the findings in this study suggest the need for future genomic studies that involve the use of PDAC organoids, to consider the genomic shift of PDAC organoids with culture. This will help to provide a more accurate interpretation of the genomic data. Furthermore, the genomic instability in PDAC organoids may not be limited to the shift in copy number profiles: It may also impact other forms of genomic variations, such as single nucleotide variations (SNVs) and other structural variants. However, the detection of SNVs from scWGS data remains challenging. This is mainly due to sequencing cost, limitations in whole genome amplification chemistry, and the availability of suitable analysis algorithms. For example, in this study, we used the multiple displacement amplification method, which introduces amplification artifacts across the genome, and the commonly used mutation detection algorithm like GATK Mutect2^39^ will detect these artifacts as SNVs^40^. Hence, improving genome amplification chemistry to minimize artifacts and developing algorithms that can address these amplification artifacts is needed for future scWGS studies. On another note, unlike copy number quantification, SNV analysis typically requires a read coverage of >30×, and the sequencing cost of ~1000 cells will be very high to achieve such coverage. Alternatively, if we assume that the clones with similar copy number profiles have similar genomic variations, then we can combine their sequencing data to increase the read depth, which then enables the SNV analysis. This approach has been tested previously with some success^41^.

Future single-cell functional genomic studies are needed to elucidate the mechanistic insights behind the growth advantage of certain clones and the genomic instability within PDAC organoids. Some groups have established the technology to sequence both genomic DNA and mRNA of the same cell^42–45^; however, currently, no commercially available technology enables the mass adaptation of such assays yet. Alternatively, considering the significant correlation between CNV and gene expression regulation (Fig. 4), we can perform scWGS and single-cell RNA-seq independently on the same organoid line, and potentially match the copy number profile of a given clone to the corresponding gene expression profile, which then allows us to derive the key regulators of the observed genomic variations. On a different note, inference of copy number profiles from RNA-seq data has been reported in multiple studies^46–49^. However, as shown in the CNV and RNA-seq correlation in Fig. 4a, the gene expression profile has much higher variations across the genome when compared to the CNV counterpart; hence, inference from RNA-seq data may lead to less accurate quantification of copy number profiles.

## Methods

### PDAC organoid culture and immunostaining

hPT1 and hPT2 PDAC organoids were derived from the primary tumors of two independent patients. The hPT1 organoids were acquired from the Barts Pancreas Tissue Bank at passage number 5 with the organoid ID B01P0735BOR (2018/14/FSU/JI/P/Organoids). The hPT2 organoids were acquired from ATCC, as part of the Human Cancer Models Initiative, at passage number 15, with the organoid name HCM-CSHL-0092-C25. The ethical approvals and the patient informed consent for these organoids were acquired by the respective sources, i.e., the Barts Pancreas Tissue Bank for hPT1 and the Cold Spring Harbor Laboratory for hPT2. The passage number listed in the manuscript represents the number of passages performed within our lab upon the first thaw. For example, hPT1 P3 in Fig. 2a was passaged three times in our lab. Maintenance of PDAC organoid culture was performed following the Tuveson Laboratory Murine and Human Organoid Protocols (http://tuvesonlab.labsites.cshl.edu/wp-content/uploads/sites/49/2018/06/20170523_OrganoidProtocols.pdf), which were kindly compiled based on that lab’s previous studies^2,3^. Both hPT1 and hPT2 organoids have a “cystic” morphology (Fig. 1a), where a distinct lumen can be observed within each organoid. Hence, as suggested by the Tuveson Laboratory Murine and Human Organoid Protocols, the organoids were passaged through mechanical dissociation, where the isolated organoids were vigorously triturated using a P1000 tip against the bottom of the centrifuge tube at least 20 times. This will ensure the shearing of the organoids into organoid fragments and the homogeneous mixing of the organoid fragments suspension. The organoids were passaged at a 1:2 ratio weekly; for example, if the original culture consisted of two 50 µL Matrigel domes, the passaged organoid fragments will be distributed into four 50 µL Matrigel domes. For immunostaining, PDAC organoids within the Matrigel (Corning) were fixed in 4% formaldehyde (EMS) for 15 min, permeabilized by 0.5% Triton-X (Sigma) for 10 min, blocked by 5% bovine serum albumin (VWR), and incubated overnight in the primary antibodies against pan-Keratin (Cell Signaling, #4545) and EpCAM (Abcam, #ab71916), both at 1:500 dilution. DNA was stained with 2 µg/mL DAPI (Sigma) for 15 min. Confocal imaging was done using a Zeiss LSM 880 system with a 63×/1.4 NA oil-immersion objective. Brightfield imaging was performed using an Olympus IX71 with a digital sCMOS camera (Prime 95B, Photometrics) and a 10×/0.3 NA objective.

### Single nucleotide polymorphism microarray

DNA isolation was performed as suggested in the Tuveson Laboratory Murine and Human Organoid Protocols, briefly the PDAC organoids were subjected to the mechanical dissociation described above, and the Blood and Cell Culture DNA Mini Kit (QIAGEN) was used to isolate the genomic DNA per manufacturer’s instruction. The isolated DNA samples were sent to the Center for Applied Genomics at the Children’s Hospital of Philadelphia for the SNP array analysis using the Infinium Global Screening Array-24 v3.0 Kit (Illumina). The chromosome copy number analysis was performed in GenomeStudio v.2.0 (Illumina) by using the cnvPartition v.3.2.1 plugin. The chromosome copy number calls were averaged every 1 Mb to derive the copy number profiles at 1 Mb resolution. Heatmaps were plotted in R by using the algorithm gtrellis v.1.28.0^50^. was used to derive their copy number profiles at 1 Mb resolution

### Single-cell whole-genome sequencing

For scWGS, to get a single-cell suspension, at least two 50 µL Matrigel domes containing the PDAC organoids were enzymatically dissociated with TrypLE Express (Thermo Fisher) following the Tuveson Laboratory Murine and Human Organoid Protocols. This resulted in a suspension of ~300,000 single cells. The single-cell suspension was thoroughly mixed, and ~20,000 cells were then processed using chromium single-cell CNV (10× Genomics) for scWGS. Briefly, this technology employs the droplet microfluidic method and the whole-genome multiple displacement amplification method. The resulting libraries of amplified DNA were subjected to 150-bp paired-end sequencing with a Novaseq 6000. The numbers of reads for the samples were approximately 180, 140, 420, and 530 million reads for hPT1 P3, hPT1 P14, hPT2 P6, and hPT2 P16, respectively. These read amounts provide sufficient read depth for the chromosome copy number quantification, per the manufacturer’s recommendation.

### Bioinformatics of the scWGS data

To derive the chromosome copy number profiles, the scWGS data was analyzed using the 10× Genomics Cell Ranger DNA algorithms and visualized using the 10× Genomics Loupe scDNA Browser. The resulting copy number profiles had a resolution of 5 Mb, meaning that each chromosome was segmented into 5 Mb parts, and each segment was represented by a copy number. For the phylogenetic tree analysis, when continuous segments in a cluster had the same copy number, we merged those segments, while making sure that the merged segments could still be vertically compared to each other across all clusters. Put another way, when a cluster had different copy numbers in the segments to be merged, we shrank the number of segments until all clusters had the same copy number on the segments to be merged. We then filtered out the segments that had the same copy number across all clusters since these segments do not contribute to the heterogeneity of the cell clusters or the construction of the phylogenetic tree. In this way, we reduced the number of unnecessary segments. Next, we used PAUP^29^ to derive maximum parsimony phylogenetic trees for the early passage clusters: hPT1 P3 clusters, called T1, and hPT2 P6 clusters, called T2, respectively. Both T1 and T2 are rooted to a copy number neutral diploid genome. Under the criterion of maximum parsimony, PAUP renders a phylogenetic tree that has a minimum number of changes of copy number states for each merged segment, along with the copy number states for all the internal nodes on the tree. The distance between each cluster and the root was calculated by summing the length of the edges along the path from the root to the cluster. To calculate the length of an edge, we first summed over the length of all segments whose copy number was different between the parent and the child nodes that the edge connects. We then divided the sum by 1 Mb. The resulting number, i.e., the copy number change measured in megabases, represents the length of this edge. We then attached the later passage clusters in hPT1 P14 to T1 through the minimal distance pairings as described in the following manner: Let set A be the clusters in hPT1 P14 that have not yet been placed on T1. Let set B be the clusters already placed on T1. We calculated the pairwise distance between all elements in A and all elements in B. The pairwise distance was calculated in the same way as calculating the length of an edge mentioned above. Suppose pair (x, y), in which x is from A, and y is from B, has the smallest distance. We attached x to y so that x becomes a daughter node of y. Since x is attached to T1, it is no longer an element of A but becomes an element of B. We thus update sets A and B accordingly. This whole process was iterated until set A becomes empty and all clusters in hPT1 P14 are attached to T1. We repeated the whole process to attach the clusters in hPT2 P16 to T2. The resulting trees T1 and T2 are the final phylogenetic tree containing both P3 and P14 for hPT1, and P6 and P16 for hPT2. In summary, we divided the construction of the phylogenetic tree into two steps in which the first step is to construct the maximum parsimony tree from the clusters in hPT1 P3, and the clusters in hPT2 P6, whereas the second step is to attach the clusters in P14 to the tree for hPT1, and the clusters in P16 to the tree for hPT2, respectively. This way, the tree honors the different passage numbers, i.e., culturing time, of hPT1 P3 and P14, as well as hPT2 P6 and P16. The phylogenetic trees were plotted using ggtree v.3.4.0^51^ and treeio v.1.20.0^52^. Heatmap visualization of the genomic data was done using gtrellis v1.28.0^50^. The t-SNE^53^ and spectral clustering were done using the TSNE and SpectralClustering functions, respectively, in Python. The hierarchical clustering was done using the heatmap.2 function from ggplots v.3.1.3^54^ in R.

### RNA-seq sample preparation and analysis

RNA isolation was performed as suggested in the Tuveson Laboratory Murine and Human Organoid Protocols, briefly the culture media was aspirated away, and the Matrigel domes were dissolved into TRIzol Reagent (Thermo Fisher), followed by the RNA isolation protocol recommended by the manufacturer. Libraries for RNA-seq were made with the NEBNext Ultra II Directional RNA Library Prep Kit for Illumina per the manufacturer’s instructions, followed by 150-bp paired-end sequencing with the Novaseq 6000 at Florida State University’s Translational Science Laboratory, resulting in ~10,000,000 reads for each sample. Reads per kilobase million for each gene were calculated by normalization of the read of each gene by the sample’s total read count (in millions) and by the gene length (in kilobases). The gene expression profile was plotted in R by using the algorithm gtrellis v.1.28.0^50^. GSEA^31,32^ of the regulated genes were performed using the “hallmark” gene sets of the human molecular signatures database (MSigDB)^33^. As suggested by the GSEA guideline, a false discovery rate < 25% was used as the threshold to select significant enrichment.

### Reporting summary

Further information on research design is available in the Nature Research Reporting Summary linked to this article.

## Supplementary information

Supplementary Figures

Supplementary Datasets

Reporting Summary

## Data Availability

The SNPa, RNA-seq, and scWGS data are available through European Genome-Phenome Archive (EGAD00001009741). Access to the data will be subjected to a Data Access Agreement.

## Code availability

The genomic data analyses in this paper use the following software: GenomeStudio v2.0, cnvPartition v.3.2.1, Cell Ranger DNA v1.1, Loupe scDNA Browser v.1.1, Phylogenetic Analysis Using Parsimony v.4.0a, gtrellis v.1.28.0, ggtree v.3.4.0, treeio v.1.20.0, ggplots v.3.1.3, GSEA 4.3.0. The script used for the phylogenetic tree analysis is available at https://github.com/compbiofan/ITH_pancreatic_organoids.git. There is no restriction to accessing the script.
