# Supplementary material for: Genomic heterogeneity in pancreatic cancer organoids and its stability with culture
Source: NPJ Genom Med. 2022 Dec 19;7:71. doi: 10.1038/s41525-022-00342-9 (PMC9763422; doi:10.1038/s41525-022-00342-9)
Supplement: Supplementary file 1 — Supplementary Figures [file 41525_2022_342_MOESM1_ESM.pdf]

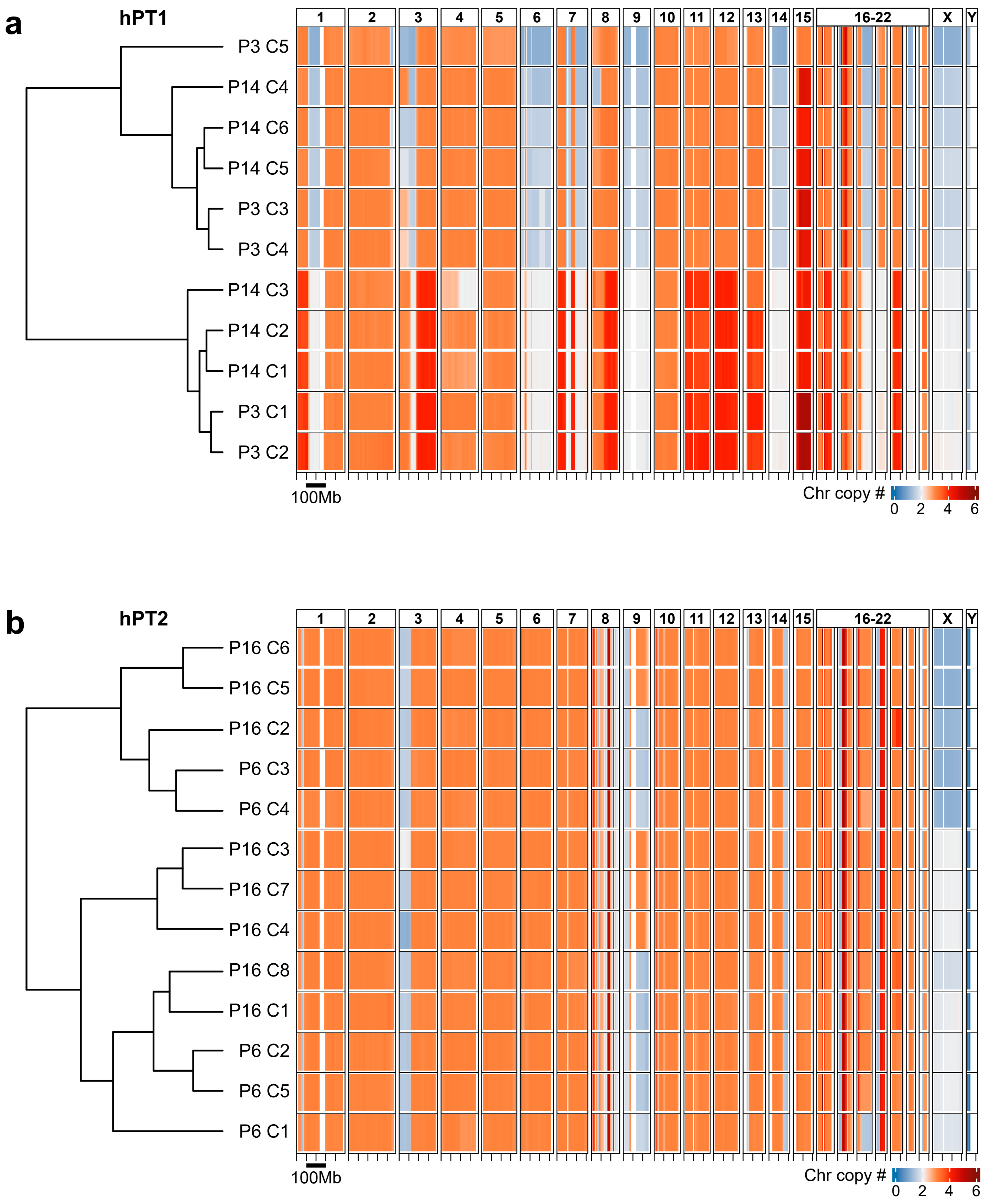

a
hPT1
P3 C5
P14 C4
P14 C6
P14 C5
P3 C3
P3 C4
P14 C3
P14 C2
P14 C1
P3 C1
P3 C2
1 2 3 4 5 6 7 8 9 10 11 12 13 14 15 16-22 X Y
100Mb
Chr copy #
0 2 4 6
b
hPT2
P16 C6
P16 C5
P16 C2
P6 C3
P6 C4
P16 C3
P16 C7
P16 C4
P16 C8
P16 C1
P6 C2
P6 C5
P6 C1
1 2 3 4 5 6 7 8 9 10 11 12 13 14 15 16-22 X Y
100Mb
Chr copy #
0 2 4 6

Supplementary Figure 1. Hierarchical clustering confirms the correlations between the sub-population clusters suggested by the phylogenetic tree analysis. The single-cell chromosome copy number data of the clusters identified in Figure 2 were averaged and their correlation was elucidated through hierarchical clustering. The distance between each cluster was calculated using the Canberra distance method. (**a**) Similar to the hPT1 phylogenetic tree in Figure 3A, the hPT1 dendrogram also reveals two distinct groups of clusters, where P3 C1-2 are closest to P14 C1-3 and P3 C3-5 are closest to P14 C4-6. (**b**) The hPT2 dendrogram shows three groups of clusters: the first one consists of P6 C1-2, C5, and P16 C1, C8, the second one consists of P16 C3-4, C7, and the third one consists of P6 C3-4, and P16 C2, C5-6. Again, this generally resembles the phylogenetic tree of hPT2 in Figure 3B.

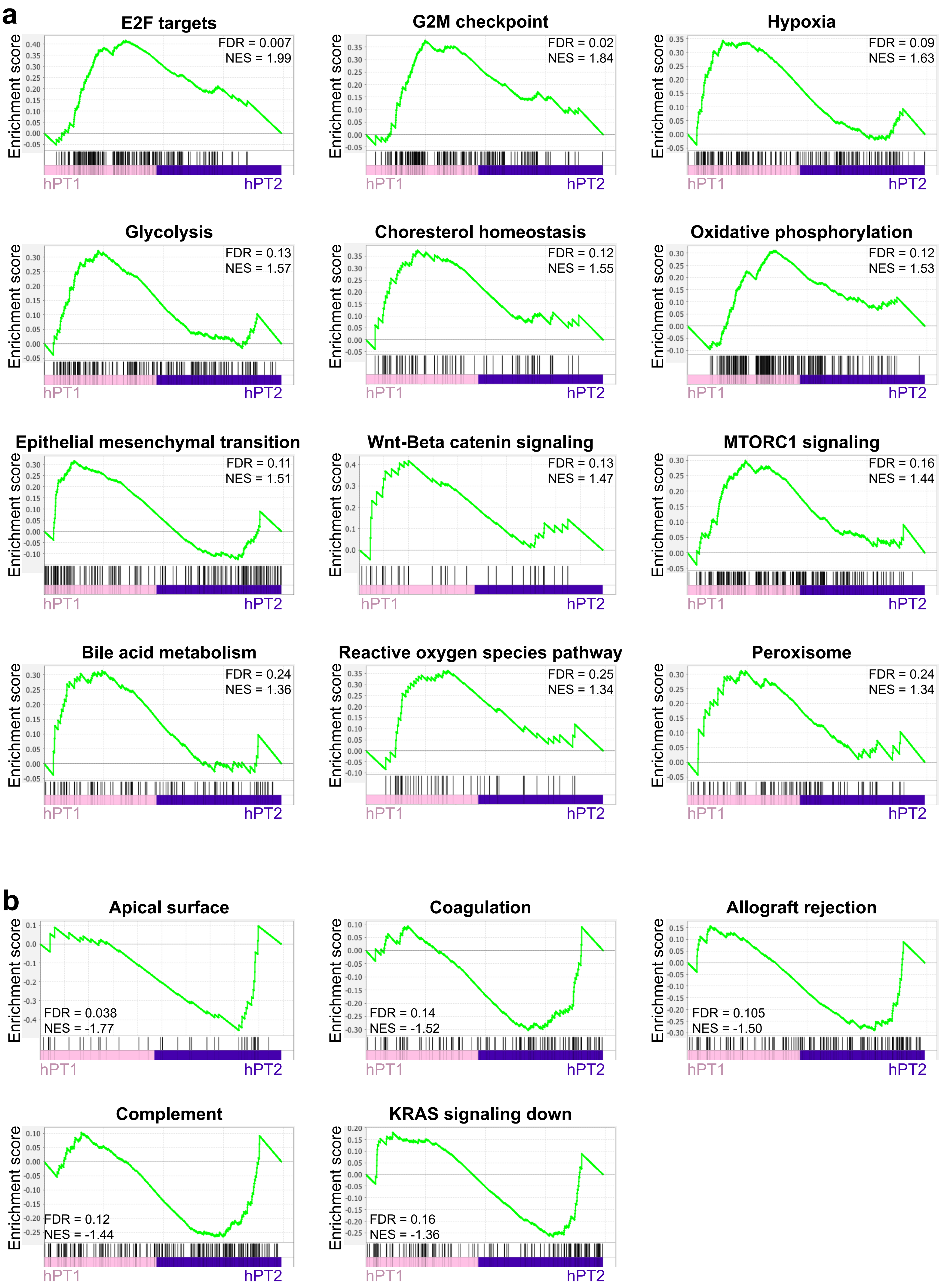
a
E2F targets
FDR = 0.007
NES = 1.99
G2M checkpoint
FDR = 0.02
NES = 1.84
Hypoxia
FDR = 0.09
NES = 1.63
Glycolysis
FDR = 0.13
NES = 1.57
Choresterol homeostasis
FDR = 0.12
NES = 1.55
Oxidative phosphorylation
FDR = 0.12
NES = 1.53
Epithelial mesenchymal transition
FDR = 0.11
NES = 1.51
Wnt-Beta catenin signaling
FDR = 0.13
NES = 1.47
MTORC1 signaling
FDR = 0.16
NES = 1.44
Bile acid metabolism
FDR = 0.24
NES = 1.36
Reactive oxygen species pathway
FDR = 0.25
NES = 1.34
Peroxisome
FDR = 0.24
NES = 1.34
b
Apical surface
FDR = 0.038
NES = -1.77
Coagulation
FDR = 0.14
NES = -1.52
Allograft rejection
FDR = 0.105
NES = -1.50
Complement
FDR = 0.12
NES = -1.44
KRAS signaling down
FDR = 0.16
NES = -1.36
Enrichment score
hPT1
hPT2

Supplementary Figure 2. Gene set enrichment analysis (GSEA) of the hPT1 and hPT2 RNA-seq data. GSEA was performed on the hPT1 and hPT2 RNA-seq data using the "hallmark" gene sets of the human molecular signatures database (MSigDB). A positive enrichment score suggests an enrichment of a gene set driven by the genes upregulated in hPT1, while a negative enrichment score suggests an enrichment of a gene set driven by the genes upregulated in hPT2. False discovery rate (FDR) < 25% was used as the threshold to select significant enrichment. (**a**) GSEA identifies 12 gene sets that are significantly enriched in hPT1, and (**b**) 5 gene sets that are significantly enriched in hPT2.
