# Supplementary material for: Genomic heterogeneity in pancreatic cancer organoids and its stability with culture
Source: NPJ Genom Med. 2022 Dec 19;7:71. doi: 10.1038/s41525-022-00342-9 (PMC9763422; doi:10.1038/s41525-022-00342-9)
Supplement: Supplementary file 3 — Reporting Summary [file 41525_2022_342_MOESM3_ESM.pdf]

nature portfolio

Corresponding author(s): Jerome Irianto

Last updated by author(s): 11/30/2022

Reporting Summary

Nature Portfolio wishes to improve the reproducibility of the work that we publish. This form provides structure for consistency and transparency in reporting. For further information on Nature Portfolio policies, see our Editorial Policies and the Editorial Policy Checklist.

Statistics

For all statistical analyses, confirm that the following items are present in the figure legend, table legend, main text, or Methods section.

n/a	Confirmed	
☒	☐	The exact sample size (*n*) for each experimental group/condition, given as a discrete number and unit of measurement
☒	☐	A statement on whether measurements were taken from distinct samples or whether the same sample was measured repeatedly
☒	☐	The statistical test(s) used AND whether they are one- or two-sided<br>*Only common tests should be described solely by name; describe more complex techniques in the Methods section.*
☒	☐	A description of all covariates tested
☒	☐	A description of any assumptions or corrections, such as tests of normality and adjustment for multiple comparisons
☒	☐	A full description of the statistical parameters including central tendency (e.g. means) or other basic estimates (e.g. regression coefficient) AND variation (e.g. standard deviation) or associated estimates of uncertainty (e.g. confidence intervals)
☒	☐	For null hypothesis testing, the test statistic (e.g. *F*, *t*, *r*) with confidence intervals, effect sizes, degrees of freedom and *P* value noted<br>*Give P values as exact values whenever suitable.*
☒	☐	For Bayesian analysis, information on the choice of priors and Markov chain Monte Carlo settings
☒	☐	For hierarchical and complex designs, identification of the appropriate level for tests and full reporting of outcomes
☒	☐	Estimates of effect sizes (e.g. Cohen's *d*, Pearson's *r*), indicating how they were calculated

Our web collection on statistics for biologists contains articles on many of the points above.

Software and code

Policy information about availability of computer code

Data collection	n/a
Data analysis	GenomeStudio v2.0 (Illumina): Software was used to analyze the data produced by the SNP microarray.<br>cnvPartition v.3.2.1 (Illumina): A plugin for GenomeStudio that was used to derive the copy number calls from the SNP microarray data.<br>Cell Ranger DNA v1.1 (10X Genomics): Software was used to derive chromosome copy number profiles from the single-cell whole genome sequencing data.<br>Loupe scDNA Browser v.1.1 (10X Genomics): Software was used to visualize the single cell whole genome sequencing data analysis from Cell Ranger DNA.<br>Phylogenetic Analysis Using Parsimony v.4.0a: Software was used to perform maximum parsimony analysis to derive the phylogenetic trees.<br>gtrellis v.1.28.0: Software was used to visualize copy number profiles in heatmap format.<br>ggtree v.3.4.0: Software was used to visualize phylogenetic trees.<br>treeio v.1.20.0: Software was used to visualize phylogenetic trees.<br>ggplots v.3.1.3: Software was used to perform the hierarchical clustering analysis.<br>GSEA 4.3.0: Software was used to perform the gene set enrichment analysis.<br><br>The script used for the phylogenetic tree analysis is available in https://github.com/compbiofan/ITH_pancreatic_organoids.git. There is no restriction to access the script.

For manuscripts utilizing custom algorithms or software that are central to the research but not yet described in published literature, software must be made available to editors and reviewers. We strongly encourage code deposition in a community repository (e.g. GitHub). See the Nature Portfolio guidelines for submitting code & software for further information.

Data

Policy information about availability of data

All manuscripts must include a data availability statement. This statement should provide the following information, where applicable:

- Accession codes, unique identifiers, or web links for publicly available datasets
- A description of any restrictions on data availability
- For clinical datasets or third party data, please ensure that the statement adheres to our policy

The SNPa, RNA-seq, and scWGS data are available through European Genome-Phenome Archive (EGAD00001009741). Access to the data will be subjected to a Data Access Agreement.

Human research participants

Policy information about studies involving human research participants and Sex and Gender in Research.

Reporting on sex and gender	n/a
Population characteristics	n/a
Recruitment	n/a
Ethics oversight	n/a

Note that full information on the approval of the study protocol must also be provided in the manuscript.

Field-specific reporting

Please select the one below that is the best fit for your research. If you are not sure, read the appropriate sections before making your selection.

☒ Life sciences ☐ Behavioural & social sciences ☐ Ecological, evolutionary & environmental sciences

For a reference copy of the document with all sections, see nature.com/documents/nr-reporting-summary-flat.pdf

Life sciences study design

All studies must disclose on these points even when the disclosure is negative.

Sample size	2 patient-derived pancreatic cancer organoids.
Data exclusions	no data exclusions
Replication	no replication
Randomization	no randomization
Blinding	no blinding

Reporting for specific materials, systems and methods

We require information from authors about some types of materials, experimental systems and methods used in many studies. Here, indicate whether each material, system or method listed is relevant to your study. If you are not sure if a list item applies to your research, read the appropriate section before selecting a response.

Materials & experimental systems

n/a	Involved in the study
☐	☒ Antibodies
☐	☒ Eukaryotic cell lines
☒	☐ Palaeontology and archaeology
☒	☐ Animals and other organisms
☒	☐ Clinical data
☒	☐ Dual use research of concern

Methods

n/a	Involved in the study
☒	☐ ChIP-seq
☒	☐ Flow cytometry
☒	☐ MRI-based neuroimaging

Antibodies

Antibodies used	pan-Keratin (C11, Cell Signaling, #4545), EpCAM (polyclonal, Abcam, #ab71916)
Validation	pan-Keratin (#4545) has been validated in the company's website through western blotting, immunohistochemistry, immunofluorescence, and flow cytometry. It has also been used in a total of 88 publications. The location of the staining in the manuscript is also as expected.<br><br>EpCAM (#ab71916) has been validated in the company's website through western blotting, immunohistochemistry, and immunofluorescence. It has also been used in a total of 128 publications. The location of the staining in the manuscript is also as expected.

Eukaryotic cell lines

Policy information about cell lines and Sex and Gender in Research

Cell line source(s)	hPT1 organoids were obtained from the Barts Pancreas Tissue Bank, with the organoid ID B01P0735BOR (2018/14/FSU/JI/P/Organoids).<br>hPT2 organoids were acquired from ATCC, as part of the Human Cancer Models Initiative, with the organoid name HCM-CSHL-0092-C25.
Authentication	hPT1 was authenticated by the Tissue Bank prior to shipment. hPT2 was authenticated by ATCC prior to shipment
Mycoplasma contamination	all cells were tested negative for mycoplasma, by using a PCR based testing.
Commonly misidentified lines<br>(See ICLAC register)	n/a
